# Flow Diverter Device-Assisted Coiling Treatment for Cerebral Blister Aneurysm: A Single-Center Study

**DOI:** 10.3390/brainsci13030435

**Published:** 2023-03-03

**Authors:** Wei Feng, Xinhua Tian, Junlong Kang, Zhaowei Han, E Chen

**Affiliations:** Department of Neurosurgery, Zhongshan Hospital Xiamen University, Xiamen 361004, China

**Keywords:** flow diverter device, blood blister-like aneurysm, ruptured aneurysm, subarachnoid hemorrhage, interventional neurosurgery

## Abstract

Although endovascular treatment is a promising approach, blood blister-like aneurysms (BBAs) still present treatment challenges. This study aimed to assess the effectiveness and safety of flow diverter device-assisted coiling (FDDAC) for the treatment of BBAs, which are broad based and friable with a high rebleeding risk. Eight patients (five females and three males) who presented with subarachnoid hemorrhages (SAH) due to BBA ruptures between May 2020 and May 2022 were retrospectively enrolled. All patients were treated by flow diverter device (Tubridge) adjunctive coil embolization using a semi-deploying technique. The demographic information, angiographic data, interval between admission and treatment, materials, therapy, clinical outcomes (including periprocedural and intraprocedural mortality and morbidity), and follow-up results of all patients were reviewed. The mean age of the patients with BBAs was 48.5 years (range 31–62 years); aneurysm sizes ranged from 2.2 × 1.7 mm to 4.6 × 3.2 mm, and the median Hunt–Hess score was 3. All aneurysms were completely closed at follow-up, and all 8 patients had excellent clinical outcomes (modified Rankin scores = 0–2) at discharge. Angiograms showed complete aneurysm occlusion after 6 months to 1 year. In addition, there were no cases of re-rupture, re-treatment, or recurrence of the aneurysms. FDDAC is safe to use in patients with BBAs and provides an alternative treatment option for this disease.

## 1. Introduction

Blood blister-like aneurysms (BBAs) are fragile, rare, and small vascular outpouchings that arise from thin-walled, broad-based, non-branching vessels and lack an identifiable neck [1]. The most common location of BBAs is the supraclinoid segment of the internal carotid artery (ICA) [2].

BBAs have histopathological features that distinguish them from saccular aneurysms. They behave as pseudoaneurysms, are composed of a thin layer of adventitia, fibrous tissue, and blood clots, and lack both a media focal arterial wall and internal elastic lamina. Such a composition contributes to their fragility. Therefore, BBAs are susceptible to rebleeding and regrowth [3,4].

BBAs are typically manifested as acute subarachnoid hemorrhages (SAH), which account for 6.6% of ruptured aneurysms and 0.3–1.7% of all intracranial aneurysms [5]. Treatment for SAHs is challenging and risky. The main goal is to avoid rebleeding of the ruptured aneurysm, and both the endovascular technique and open neurosurgery can achieve a good outcome. However, these two approaches are difficult to implement due to the fragility of BBAs [6].

Many techniques, including clipping and wrapping, surgical clipping, suturing, bypass, trapping, or stenting with/without the assistance of coils, have been developed. However, there remains no clear consensus on the optimal treatment for BBAs [7].

The flow diverter device (FDD) has become a valuable tool for managing difficult and unruptured aneurysms, such as large or giant ICA aneurysms, and is increasingly employed in the treatment of other aneurysms, especially BBAs at the acute hemorrhage stage [8]. Single or multiple FDDs can be used to disrupt and modify blood flow inside the parent artery, thus allowing for endoluminal reconstruction. This approach has achieved positive results [9,10]. Refinements have expanded the application of this technique, for example, the use of a stent retriever device to achieve optimal FDD placement in giant aneurysms [11].

However, BBAs require weeks or months to achieve total occlusion and enlargement. Despite FDD placement, fatal rebleeding has been reported in some patients [12,13].

It is likely that re-rupturing after PED placement may be caused by failure to exclude an aneurysm from blood flow, an effect that is successfully produced by microsurgical clipping or direct coil embolization. Hence, treatment of ruptured BBAs by individual FDD placement remains controversial [14].

The current study investigated the ability of FDDAC by using the semi-deploying technique to achieve both rapid aneurysm occlusion and excellent follow-up results. The aim of this study was to assess the effectiveness, safety, and midterm follow-up results of FDDAC for BBAs from the perspective of a single-center experience.

## 2. Materials and Methods

The current study included all patients diagnosed with BBAs who were acutely treated by FDDAC in our hospital from May 2020 to May 2022. Patient demographic data (age and sex), presenting symptoms (Hunt–Hess score and Glasgow coma score), radiological images (CT angiography (CTA) and digital subtraction angiography [DSA]), operative reports, (including FDD type, antiplatelet agents, complications, and post-procedural angiographic outcomes), and follow-up results were recorded and reviewed.

### 2.1. Study Population

Eight patients (five females and three males; mean age = 48.5 years; range = 31–62 years old) were retrospectively enrolled.

All cases with SAHs due to a ruptured intracranial aneurysm of size ranging from 2.2 × 1.7 mm to 4.6 × 3.2 mm were examined by enhanced CT. All BBAs were located in the supraclinoid segment of the ICA, with 3 on the left ICA and 5 on the right. Initial Hunt–Hess Grades were 2 in two patients, 3 in five patients, and 4 in one patient (Table 1).

### 2.2. Description of the Tubridge FD and Endovascular Treatment

In all cases, the Tubridge, a new type of FDD developed by the MicroPort Medical Company (Shanghai, China), was the flow diverter of choice. The Tubridge FD is a self-expanding, braided, stent-like device with flared ends, which has varying structures based on its diameter. The small FD has a diameter of <3.5 mm and consists of 2 radio-opaque, platinum–iridium, and 46 nitinol microfilaments to improve the visualization of length and diameter during placement procedures. The large FD has a diameter of ≥3.5 mm and consists of 2 radio-opaque and 62 nitinol microfilaments. The Tubridge FD gives higher metal coverage (30–35%) than conventional stents at the aneurysmal neck [15]. The midpoint of the Tubridge FD is marked, and the FDD can be retracted before being released up to that point [16].

After an extensive discussion on each case, the open endovascular and cerebrovascular neurosurgical teams made the decision to use an FD stent by taking into account all possible treatment options. The endovascular procedure is normally performed using a triaxial (guiding, intermediary, and microcatheters) catheter technique. All patients treated with FDDAC were given aspirin (300 mg) and clopidogrel (300 mg) 4–8 h prior to endovascular treatment. After general anesthesia, all procedures were conducted in a catheter room. Heparin was routinely administered during the procedure. An 8-French (F) femoral artery short sheath was used to canalize the right femoral arteries. DSA and 3D rotational angiographic images were obtained to verify the BBA size, formation, and location as well as the parent artery diameter. A 6-F Flexor Check-Flointroducer Set (COOK, Indiana, USA) was introduced at the proximal ICA via the left 8-F femoral artery short sheath. A 5-F Navein guiding catheter (Medtronic, California, USA) was placed at the petrosal segment of the parent ICA through the 6-F Flexor Check-Flointroducer Set. A Fastrack microcatheter (MicroPort, Shanghai, China) was positioned at the M2 segment of the middle cerebral artery via the Navien guiding catheter. The coiling microcatheter (Echelon 10, Medtronic, MN, USA) was brought up and placed into the aneurysms over the 6-F Flexor Check-Flointroducer Set. A semi-deploying technique was then employed for FDDAC therapy. Briefly, 1 or 2 coil loops were first advanced into the aneurysms through the Echelon 10 microcatheter, and the FDD gently semi-deployed under fluoroscopic guidance in order to partially expand and cover the aneurysm neck. The Echelon 10 microcatheter was partly stuck between the FDD and parent arteries. The coils were carefully embolized within the BBAs, and the FDD was further deployed if the coils bulged into the ICA. After completing the embolization, the Echelon 10 microcatheter was slowly removed and the FDD was fully deployed. The expansion of the FDD was verified by Dyna CT computed tomography angiography and fluoroscopy. Dual antiplatelet therapy was maintained for 6 months after the procedures followed by indefinite aspirin monotherapy (Table 2). In one case, the patient did not take antiplatelet drugs for a sufficient duration (only 4 h) due to the operation, necessitating the intravenous administration of tirofiban during the endovascular procedure to enhance the antiplatelet effect and prevent intraoperative and post-operative arterial thrombosis.

Clinical follow-ups were conducted at 2 weeks, 3 months, and 6 months to 1 year. Imaging follow-ups were carried out prior to discharge in severe patients and between 6 months and one year post-discharge. Imaging outcomes were dichotomized into complete or incomplete occlusion according to the follow-up catheter angiograms. The modified Raymond–Roy occlusion classification was used to grade the occlusion of endovascularly treated intracranial aneurysms. Modified Rankin scale (mRS) scores of 0–2 were regarded as excellent clinical outcomes.

## 3. Results

A technically successful outcome of the procedure was achieved in all cases, and excellent clinical outcomes (modified Rankin score = 0–2) were observed in all eight patients at discharge. Three patients had an mRS of 0, four had an mRS of 1, and one had an mRS of 2. Early angiograms from the 2-week follow-ups after initial treatment revealed complete resolution of the aneurysms in six patients, while 3-month follow-up angiograms demonstrated complete resolution in seven patients. Complete occlusion of the aneurysms was observed in all eight patients at the 6-months to 1-year follow-ups. There were no reports of re-rupture, re-treatment, or recurrence of the aneurysms (Table 3).

## 4. Typical Case

**Figure 1:**
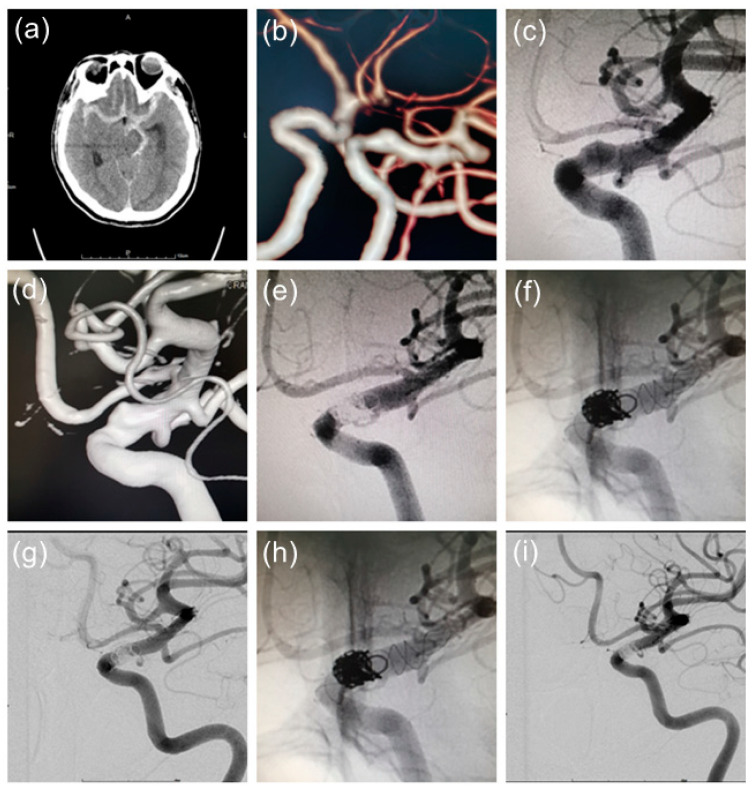
Thirty-six-year-old male presented with a severe, acute headache, accompanied by a brief period of unconsciousness, and an axial CT head showed a subarachnoid hemorrhage (**a**). A pretreatment CT angiogram exhibited an aneurysm in the clinoid segment of the left intracranial artery (**b**). DSA examination and 3D reconstruction cleared the ruptured blood blister-like aneurysm (treatment angle) (**c**,**d**). During the process, the patient underwent FDD-assisted coiling via a semi-deploying method (Tubridge 3.5 × 25 mm). After treatment, the aneurysm was completely occluded (**e**,**f**). Follow-up angiograms at 3 months (**g**,**h**) and 6 months post-procedure showed complete resolution of the aneurysm with remodeling of the parent vessel (**i**).

**Figure 2:**
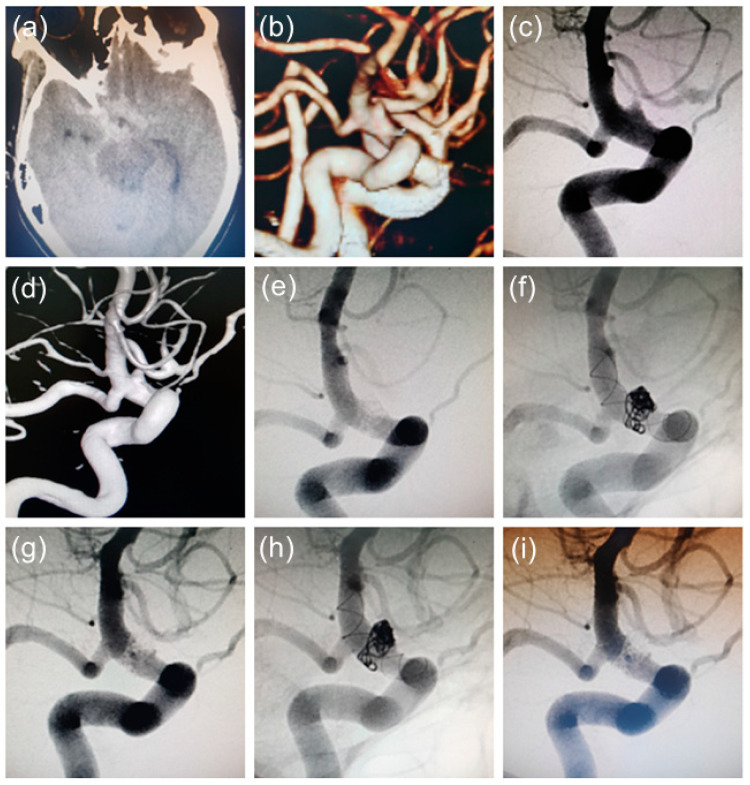
53-year-old female presented with severe headache accompanied by unconsciousness, and an axial CT head showed a subarachnoid hemorrhage (**a**). A pretreatment CT angiogram exhibited an aneurysm in the clinoid segment of the right intracranial artery (**b**). DSA examination and 3D reconstruction cleared the ruptured blood blister-like aneurysm (treatment angle) (**c**,**d**). During the process, the patient underwent FDD-assisted coiling via a semi-deploying method (Tubridge 3.5 × 20 mm). After treatment, the aneurysm was completely occluded (**e**,**f**). Follow-up angiograms at 3 months (**g**,**h**) and 6 months post-procedure showed complete resolution of the aneurysm with remodeling of the parent vessel (**i**).

**Figure 3:**
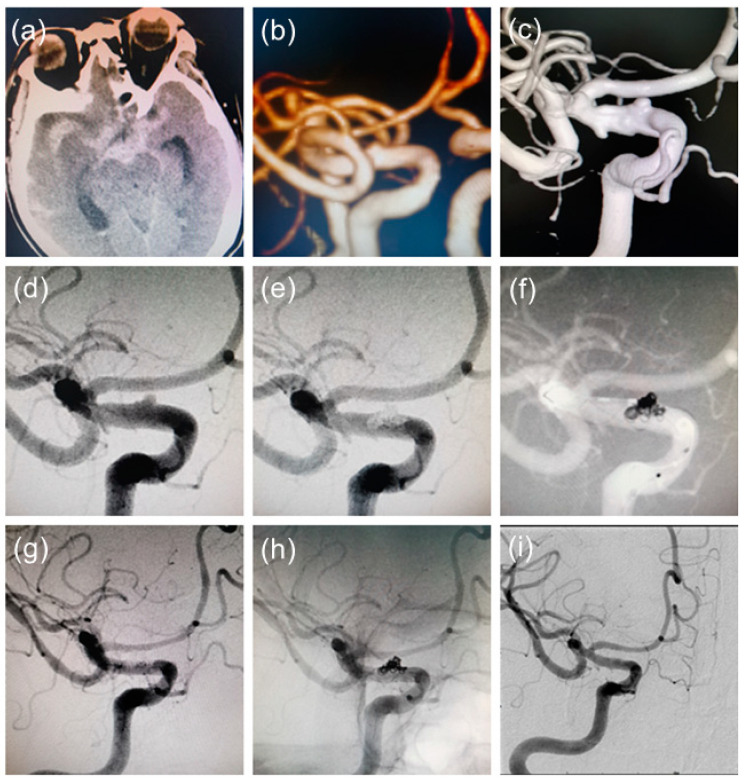
56-year-old female presented with sudden unconsciousness, and an axial CT head showed a subarachnoid hemorrhage (**a**). A pretreatment CT angiogram exhibited an aneurysm in the clinoid segment of the left intracranial artery (**b**). DSA examination and 3D reconstruction cleared the ruptured blood blister-like aneurysm (treatment angle) (**c**,**d**). During the process, the patient underwent FDD-assisted coiling via a semi-deploying method (Tubridge 4.0 × 20 mm). After treatment, the aneurysm was completely occluded (**e**,**f**). Follow-up angiograms at 3 months (**g**,**h**) and 6 months post-procedure showed complete resolution of the aneurysm with remodeling of the parent vessel (**i**).

All patients suffered from severe symptoms. The CT scan demonstrated an SAH (Figure 1a, Figure 2a and Figure 3a). The CTA exhibited an aneurysm in the clinoid segment of the intracranial artery (Figure 1b, Figure 2b and Figure 3b), and emergency surgery was initiated. DSA showed the size and location of the aneurysm (Figure 1c, Figure 2c, Figure 3c, Figure 1d, Figure 2d and Figure 3d). The patient underwent FDD-assisted coiling via a semi-deploying method in our hospital. After two microcatheters (Fastrack microcatheter/Echelon 10 microcatheter) were introduced at the designated spot, one loop of coil was partially inserted into the BBA. Subsequently, the Tubridge (FDD) was gently semi-deployed to cover part of the aneurysm neck. Then, the first coil was completely filled in, and more loops of coil were advanced according to the need until the aneurysm was no longer visible. After that, the Tubridge was fully deployed. After treatment, the aneurysm was completely occluded (Figure 1e, Figure 2e, Figure 3e, Figure 1f, Figure 2f and Figure 3f). During the operation, angiography was performed from time to time to confirm the condition of the aneurysm. The DSA assessment conducted 3/6 months after the procedures revealed a non-stenotic intracranial artery and no recurrence of the BBA (Figure 1g–I, Figure 2g–I and Figure 3g–i).

## 5. Discussion

Despite the availability of several treatment approaches (e.g., microsurgical, endovascular, or hybrid therapy), the management of patients with BBAs remains challenging. However, a reconstructive endovascular treatment, which targets the diseased segment of the parent artery and embolizes the BBA, has been increasingly accepted due to its rapid development over the past few decades [17].

The most common endovascular approaches used for treating BBAs include stent-assisted coiling (SAC) embolization and FD stents. However, the thinness and fragility of the BBA wall means that primary or stent-assisted coiling of these wide-necked and fragile aneurysms has a high risk of intraprocedural rupture, in which case embolization may be achieved by balloon inflation, and post-operative rebleeding, especially during the process of microcatheter manipulation and coil placement [18,19].

The occlusion rates of BBAs after stent-assisted coiling are not satisfactory, producing a 33% initial occlusion after the procedure and approximately 70% at the mid-to-long-term follow-up [6]. In a study of thirty-four patients with ruptured BBAs, Lim and colleagues employed a single stent with coiling in six patients and multiple overlapping stents with/without coiling in twenty-eight patients. They found that three cases had rebleeding (9%), leading to one death (3%). Aneurysm recurrence was also detected in 25% of patients within 5 weeks [20].

A previous study retrospectively reviewed 212 BBA patients who received stent-assisted coiling and found that 64.6% were completely healed but 22.9% had recurrence [10]. A pooled analysis of 76 SAC patients conducted by Rho and co-workers showed that the rates of complete occlusion, rebleeding, recurrence, and total mortality were 54.8%, 7.94%, 24.2%, and 7.7%, respectively, after treatment [21]. It is well known that a stent can offer scaffolding for endothelialization and disrupt the blood flow at the inflow area of the aneurysm. However, a single high-porosity stent is not sufficient to prevent rebleeding and recurrence. Although overlapped stenting provides greater metal coverage at the neck of BBAs and exhibits better outcomes, the associated complication rate is still not low [22]. Because of the high recurrence rate, stent-assisted coiling embolization may not be the best approach to BBA treatment.

As reported by many previous studies, FDDs are endovascular stents that reduce hemodynamic stress on the aneurysm wall, remodel the diseased vessel segments, and preserve lumen patency [23]. They can induce intra-aneurysmal thrombosis and subsequently occlusion, which is achieved by a narrowly braided stent wall. FD stents have been used successfully in the treatment of ruptured BBAs [24,25,26,27]. A recent meta-analysis of BBAs treated with endovascular approaches, including FD and SAC, from 2009 to 2020 showed that FD had a higher long-term complete occlusion rate and lower aneurysm recanalization rate than SAC (89.26% vs. 70.26% and 4.54% vs. 25.38%, respectively) [13].

Linfante and co-workers found that 90% of patients with a ruptured BBA received adequate treatment of a single PED and concluded that this may be a durable and safe option [28]. Indeed, Zhu and colleagues found that a single PED could offer better outcomes than overlapping PEDs during a review of 165 patients with BBAs [5]. Disruption of flow to the aneurysm can induce aneurysmal shrinkage and intrasaccular thrombosis, although such a process needs weeks or months to achieve total occlusion during which there is a high re-rupture risk [12,13]. In order to decrease the risk of rebleeding and re-rupture in the acute phase, better treatment is needed. Ryan and co-workers reported complete occlusion of 31% of 16 BBAs (which were not obliterated on the day of treatment) at the three-month follow-up [29]. Other studies found that aneurysm growth and re-rupture could lead to death following a single PED deployment [30,31].

Furthermore, although intraoperative contrast stasis and reduced blood flow were identified in the aneurysm sac after PED placement, this did not indicate a reduction in pressure. Cebral and colleagues conducted computational hemodynamic research and demonstrated that an increase in intra-aneurysmal pressure after FD deployment is correlated with re-rupture [32]. These cases show that early intravascular embolization can be an effective strategy for preventing re-rupture. Thus, a therapeutic approach using coil embolization followed by PED has been recommended to reconstruct blood flow in the parent arteries [33,34,35]. The coil embolizes the aneurysm sac, loosely packs the aneurysm neck, and assists the PED in repairing the parent arteries. Hence, the parent arteries were re-established simultaneously with the embolization of the BBA. Similarly, Liu and co-workers also reported the successful treatment of single PED-assisted coil embolization in 12 patients with BBAs, in which all patients showed complete occlusion on post-operative angiographies [36]. Thus, FDDAC may be a viable therapeutic option for cerebral BBAs. It has been suggested that overlapped FDDs can be used to raise the chance of gaining an immediate BBA occlusion. However, higher complication rates have been found with multiple FDDs compared to a single FDD [37,38]. Damiano and colleagues reported that a compression of the FDD in front of the aneurysm neck was more efficacious in flow disruption than overlapped FDDs [38]. Additionally, a systematic review and meta-analysis revealed that the single FDD approach had better outcomes in patients with BBAs compared to the overlapped FDD approach (89.9% vs. 61.9%; odds ratio = 1.42) [39].

Our results are also in agreement with these findings. There are several reasons for the successful application of FD stent-assisted coiling in 10 patients with ruptured BBAs. First, the flow diverter redirects the blood flow away from the BBA and allows for parent artery reconstruction, thereby reducing the risk of rebleeding and recurrence. Following the endovascular procedure, there was no rebleeding or recurrence of the aneurysms treated by FDDAC, and no clinical complications were directly attributable to the FDD implants or coil embolization. In addition, coils within the BBA sac provide mechanical support for the thrombus in the acute stage, thus obtaining a compact embolization in the BBAs.

Midterm follow-ups in the current work indicated low re-treatment/re-rupture rates and high occlusion rates with excellent clinical outcomes in most of the patients. Nevertheless, the main concern is that the application of FDDs may lead to the need for antiplatelet agents during the acute phase. We have addressed this problem by administering dual antiplatelet therapy (300 mg aspirin and 300 mg clopidogrel) immediately before the emergency operation and 90 mg (b.i.d.) of ticagrelor plus 100 mg (q.d.) of aspirin in the following hours. The use of dual antiplatelet therapy in cases of severe rupture is still challenging and may require careful monitoring. Anti-glycoprotein IIb/IIIa antibodies have previously been used during FDD placement [40] but were not given during the current study. Comparative investigations to assess the use of this alternative form of antiplatelet therapy in the context of FDDAC treatment are required.

It should be noted that some intra- and periprocedural complications are associated with FDDs, such as side-branch occlusion, intraoperative rebleeding, stent thrombosis, post-procedural intracranial hemorrhages, and vasospasm. Some of these outcomes may be partly attributable to the application of dual antiplatelet therapy, which can promote the occurrence of major bleeding [28]. Nonetheless, the high mortality and morbidity rates associated with FDD-treated BBAs were lower than those following surgery, even when dual antiplatelet therapy was administered [5]. It has been reported that the glycoprotein IIb/IIIa inhibitor, tirofiban, is safe to use along with dual antiplatelet therapy as a protocol for patients with intracranial aneurysms undergoing FDD treatment [41].

We acknowledge some limitations to the present study. This was a retrospective, observational, cohort study that involved a small sample size. Hence, the rate of complications (e.g., the rebleeding rate of FDD-treated aneurysms) may be lower. Moreover, due to the rarity of this disease, it was difficult to collect substantial data. Further variations in the surgical approach could not be considered during the current work, such as alternative approaches to antiplatelet therapy. Thus, long-term follow-up studies are required to ensure the durability of FDDAC treatment for BBAs.

## Figures and Tables

**Table 1:** Baseline, radiographic, and clinical data of BBA patients.

Patient	Age	Gender	Past Medical History	Hunt–Hess Grade	GCS Score	Location	Size (mm, Neck × Dome)
1	31	Male	Hypertension, arthrolithiasis (articular gout)	3	11	Left ICA	4.6 × 3.2
2	53	Female	Hypertension	3	10	Right ICA	3.2 × 2.3
3	56	Female	Hypertension	3	9	Right ICA	2.5 × 2.0
4	36	Male	None	2	15	Left ICA	3.4 × 2.7
5	45	Female	Hypertension	4	7	Left ICA	3.2 × 1.8
6	51	Female	Hypertension	2	15	Right ICA	2.2 × 1.7
7	54	Female	Hypertension, coronary heart disease	3	13	Right ICA	3.5 × 2.1
8	62	Male	Hypertension	3	13	Right ICA	3.8 × 2.4

**Table 2:** Details of the endovascular procedures.

Patient	Tubridge FD (mm)	Admission to Treatment (h)	Pre-Operative Antiplatelet Drugs	Post-Operative Antiplatelet Drugs	Coil
1	3.5 × 25	7	Clopidogrel (300 mg) + Aspirin (300 mg)	Ticagrelor 90 mg (b.i.d.) + Aspirin 100 mg (q.d.)	3 × 60 mm/1.5 × 20 mm/1.5 × 20 mm
2	3.5 × 20	9	Clopidogrel (300 mg) + Aspirin (300 mg)	Ticagrelor 90 mg (b.i.d.) + Aspirin 100 mg (q.d.)	2 × 40 mm/1.5 × 20 mm
3	4.0 × 20	7.5	Clopidogrel (300 mg) + Aspirin (300 mg)	Ticagrelor 90 mg (b.i.d.) + Aspirin 100 mg (q.d.)	1.5 × 40 mm/1 × 20 mm
4	4.5 × 25	8	Clopidogrel (300 mg) + Aspirin (300 mg)	Ticagrelor 90 mg (b.i.d.) + Aspirin 100 mg (q.d.)	2 × 40 mm/1.5 × 20 mm
5	3.5 × 15	6	Clopidogrel (300 mg) + Aspirin (300 mg)	Ticagrelor 90 mg (b.i.d.) + Aspirin 100 mg (q.d.)	1.5 × 40 mm/1.5 × 20 mm
6	4.0 × 25	10	Clopidogrel (300 mg) + Aspirin (300 mg)	Ticagrelor 90 mg (b.i.d.) + Aspirin 100 mg (q.d.)	1.5 × 20 mm/1 × 10 mm
7	4.0 × 20	9	Clopidogrel (300 mg) + Aspirin (300 mg)	Ticagrelor 90 mg (b.i.d.) + Aspirin 100 mg (q.d.)	2 × 40 mm/1.5 × 20 mm
8	3.5 × 15	7.5	Clopidogrel (300 mg) + Aspirin (300 mg)	Ticagrelor 90 mg (b.i.d.) + Aspirin 100 mg (q.d.)	2 × 60 mm/1.5 × 20 mm

**Table 3:** Clinical and angiographic data during the follow-up period.

Patient	Raymond Roy Scale at the 2-Week Follow-Up	mRS Score at Discharge	Raymond Roy Scale at the 3-Month Follow-Up	Raymond Roy Scale at the 6-Month to 1-Year Follow-Up
1	1	0	1	1
2	2	1	1	1
3	1	1	1	1
4	1	0	1	1
5	2	2	2	1
6	1	0	1	1
7	1	1	1	1
8	1	1	1	1

## Data Availability

Not applicable.
